# Hippocampal and cortical communication around micro-arousals in slow-wave sleep

**DOI:** 10.1038/s41598-019-42100-5

**Published:** 2019-04-10

**Authors:** Gustavo Zampier dos Santos Lima, Bruno Lobao-Soares, Gilberto Corso, Hindiael Belchior, Sergio Roberto Lopes, Thiago de Lima Prado, George Nascimento, Arthur Cavalcanti de França, John Fontenele-Araújo, Plamen Ch. Ivanov

**Affiliations:** 1 Escola de Ciências e Tecnologia, Universidade Federal do Rio Grande do Norte, Natal, RN Brazil; 2 Departamento de Biofísica e Farmacologia, Universidade Federal do Rio Grande do Norte, Natal, RN Brazil; 3 Departamento de Fisiologia e Comportamento, Universidade Federal do Rio Grande do Norte, Natal, RN Brazil; 4 Faculdade de Ciências da Saúde do Trairí, Universidade Federal do Rio Grande do Norte, Natal, RN Brazil; 5 Departamento de Física, Universidade Federal do Paraná, Curitiba, PR Brazil; 6 Instituto de Engenharia, Ciência e Tecnologia, Universidade Federal dos Vales do Jequitinhonha e Mucuri, Janaúba, MG Brazil; 7 Departamento de Engenharia Biomédica, Universidade Federal do Rio Grande do Norte, Natal, RN Brazil; 8 Instituto do Cérebro, Universidade Federal do Rio Grande do Norte, Natal, RN Brazil; 9 Keck Laboratory for Network Physiology, Department of Physics, Boston University, Boston, MA, USA; 10 Division of Sleep Medicine and Harvard Medical School, Brigham and Women’s Hospital, Boston, MA, USA

## Abstract

Sleep plays a crucial role in the regulation of body homeostasis and rhythmicity in mammals. Recently, a specific component of the sleep structure has been proposed as part of its homeostatic mechanism, named micro-arousal. Here, we studied the unique progression of the dynamic behavior of cortical and hippocampal local field potentials (LFPs) during slow-wave sleep-related to motor-bursts (micro-arousals) in mice. Our main results comprised: (i) an abrupt drop in hippocampal LFP amplitude preceding micro-arousals which persisted until the end of motor-bursts (we defined as t interval, around 4s) and a similar, but delayed amplitude reduction in cortical (S1/M1) LFP activity occurring at micro-arousal onset; (ii) two abrupt frequency jumps in hippocampal LFP activity: from Theta (6–12 Hz) to Delta (2–4 Hz), also t seconds before the micro-arousal onset, and followed by another frequency jump from Delta to Theta range (5–7 Hz), now occurring at micro-arousal onset; (iii) a pattern of cortico-hippocampal frequency communication precedes micro-arousals: the analysis between hippocampal and cortical LFP fluctuations reveal high coherence during τ interval in a broader frequency band (2–12 Hz), while at a lower frequency band (0.5–2 Hz) the coherence reaches its maximum after the onset of micro-arousals. In conclusion, these novel findings indicate that oscillatory dynamics pattern of cortical and hippocampal LFPs preceding micro-arousals could be part of the regulatory processes in sleep architecture.

## Introduction

Sleep has been described as a crucial behavioral state to physiological regulations, including hormone secretion^1^, learning processes^2^ and immune activity^3^. In mammals, sleep is divided into two main stages, rapid eye movement (REM)/Paradoxical sleep, and slow-wave sleep (SWS). While REM sleep is characterized by some physiological similarities to waking states in cortical electroencephalogram (EEG), such as low amplitude and high-frequency oscillations, SWS is defined by a high amplitude and low-frequency oscillations^4^. The sleep-wake cycle is controlled by periodic mechanisms within 24-hour periods, and the sleep phase itself oscillates between SWS and REM in an ultradian rhythm^5,6^. These two rhythms are fundamental to the homeostatic regulation of living organisms.

The coordinated activity among many encephalic structures promotes the transition from sleep to wake states. The arousal brain system is comprised by the ascending reticular activating system (ARAS) and a descending network from the cortex to the spinal cord. ARAS emerges from neuronal nuclei located in midbrain and pons and projects to sub-cortical structures that stimulate cerebral cortex and generate fast EEG oscillations^7^. In turn, a descending network is related to motor control allowing high amplitude movements, associated with increased electromyography amplitude (EMG), which corresponds to classical motor system pathways^8^.

In particular, sleep phases can be often interrupted by small, transient micro-arousal events that naturally emerge even during deep sleep stages. They are believed to recruit, at least partially, structures involved in the global arousal process^9^. The occurrence of micro-arousals was firstly interpreted as short wakefulness intrusions into sleep^10^. Pathological micro-arousals were also related to sleep disorders such as central apneas^11–13^, in which they were effective to restore ventilation overshoots^14^. Nevertheless, the role of physiological micro-arousals remains to be fully understood^10,15^. Micro-arousals are characterized by abrupt alterations of oscillatory patterns in the LFPs, in particular, of low-frequency bands^5,16^. In humans, micro-arousals are characterized by low-voltage fast-rhythm EEG oscillations, which can be accompanied by bursts of high-amplitude delta oscillations^10^. In rodents, micro-arousals during slow-wave sleep can encompass the emergence of hippocampal theta oscillations associated with increases in the frequency of ECG and in the amplitude of EMG^17–19^, which often include rapid and transient head movements^20^. Furthermore, physiological micro-arousals are quite common during SWS. They are associated with small irregular activity (SIA) in hippocampal LFPs, which are characterized by a sharp decrease in the oscillatory amplitude^20–23^. SIA has been previously proposed as a mechanism for maintaining context memory during sleep^17^. Recordings of single units in the rodent hippocampus indicate that SIA reflects a partial arousal in response to internal/external stimuli, which allows the animal to assess whether the full awakening is warranted, without disrupting the sleep cycle. Noteworthy, other studies suggested that the hippocampus may play a pivotal role in information processing during SIA events in sleep. Thus, the description of these electrical hippocampal phenomena during SIA events indicates that this brain structure could potentially act as part of the activating arousal brain system.

A recent work^20^ examined the temporal dynamics of micro-arousal events using head-accelerometer recordings. The authors reported that accelerometer signal (Acc) can predict motor-bursts typically observed in micro-arousals, through a specific technique called recurrence plot^24^. Furthermore, micro-arousals were preceded (about 4 seconds) by a drop in the determinism of Acc (a quantifier that computes how accurate are predictions based on past trajectories^24^), as well as by a simultaneous decrease in the standard deviation of the LFPs in the CA1 area of the hippocampus.

The interplay between cortical and sub-cortical structures of the brain contribute to the generation of oscillatory patterns found during SWS^25^. Recent studies have provided electrophysiological evidence of a robust coupling between hippocampal and cortical areas during SWS^26–28^. Lately, it was shown that micro-arousals were often preceded and succeeded by periods of low LFP amplitude and low firing rate activity, both in neocortical and hippocampal networks^29^. Despite such studies have shed some light on the understanding of physiology and physiopathology of micro-arousals in sleep architecture, little is known about the relationship involving hippocampal activities, transient cortico-hippocampal communication and micro-arousals episodes during SWS.

Here we investigated the following questions: Are micro-arousal events associated with cortical and hippocampal activities modulations? Do cortical and hippocampal activities change simultaneously at micro-arousals events? Is there a typical brainwave communication between cortex and hippocampus during SWS micro-arousals? Aiming to address these questions, we used micro-electrode arrays to record hippocampal and cortical LFP activities around micro-arousal events during deep SWS in mice.

## Methods

### Ethical compliance

This study complied with all ethical regulations regarding animal experimentation. All housing, surgical, and behavioral procedures presented in this work were in accordance with the National Institutes of Health guidelines, and approved by the Edmond and Lily Safra - International Institute of Neuroscience of Natal (ELS-IINN) Ethics Committee for Animal Experimentation (protocol number 08/2010).

### Experimental Procedure

#### Animals

**Figure 1:**
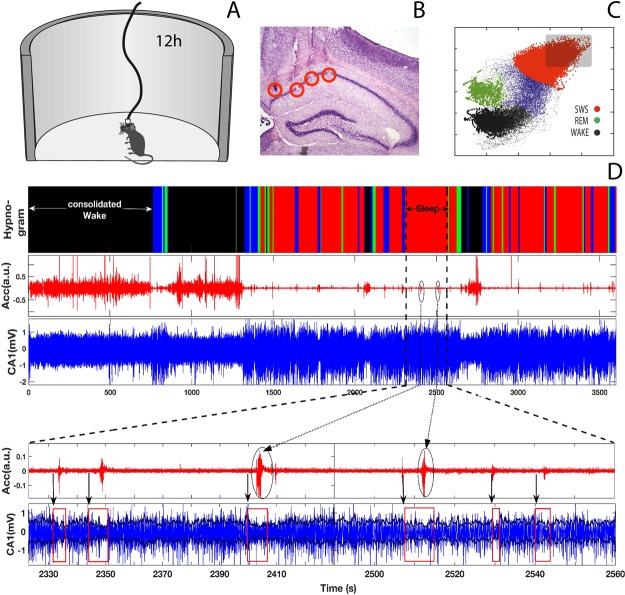
Schematic presentation of experimental procedures to record electrophysiological dynamics associated with micro-arousals. Panel (A) shows the multi-electrode arrays were implanted in cortical and hippocampal areas of the mice brain and a three-axis accelerometer was attached the amplifier headstage. Also, the raw electrophysiological signals were recorded while mice explored a circular open field. Panel (B) display the histological photograph showing typical lesions caused by electrode implantation in the mouse brain. Using the state map analysis we identify sleep stages for each mouse derived from LFP signals to identify state-map areas corresponding to different sleep-stages - deep sleep (DS), light sleep (LS), REM sleep and wake. (see Method). Sleep stage hypnogram plot was derived over time from the state map plot obtained for each animal. In Panel (D) we present the stages and associated physiological dynamics: Sleep stage hypnogram (top panel) and parallel physiological recordings derived from accelerometer (Acc, red), hippocampus (CA1, dark blue) channels indicating changes in amplitude morphology of these signals with transitions from one sleep stage to another. Data represent one-hour recordings from an individual mouse during a lights-on phase. Large-scale observation shows high amplitude for the locomotions-related Acc and EMG signals during wake and greatly reduced amplitude during light and deep sleep. In contrast, an opposite behavior is observed for the LFP signal derived from the CA1 hippocampal area: high amplitude during light and deep sleep and low amplitude during the wake. Observations at small time scales, however, reveal a multitude a short burst with smaller amplitude in the Acc locomotor activity signals (micro-arousal) that persistently occur even during SWS sleep. A magnification of a 4-min SWS period (marked by vertical dashed-lines) is shown in the bottom panels: multiple micro-arousals represented by short bursts in the Acc signals (highlighted by black ovals) are consistently associated with short intervals of significant drop in the amplitude of the hippocampus CA1 signal (highlight by red rectangles), rising the question whether these micro-arousals are of internal nature.

Five adult male C57Bl-6 mice (2–5 months) were used in this series of experiments. After surgery, animals were housed in individual home cages under 12/12 h light/dark schedule (lights on at 7 am), with food and water freely available (Fig. 1).

#### Multielectrode Implantation Surgery

Animals were surgically implanted with chronic multielectrode arrays for intracranial local field potentials recordings. We used 13 teflon-coated tungsten microwires (diameter, 50 um; interelectrode spacing, 250 um; impedance, 0.5 MOhm at 1 KHz) attached to Omnetics 16-pin connector. The arrays were implanted through a rectangular craniotomy (Coordinates from Bregma: 0.55 mm to 1.65 mm to the right hemisphere, 0.0 mm to 2.2 mm posterior) under isoflurane anesthesia. For each animal, 5 electrodes were stereotaxically positioned in the dorsal CA1 area of the hippocampus, 4 electrodes in the M1 area of the motor cortex and 4 electrodes in the S1 area of the somatosensory cortex (1.5 deep into the brain). Three screws attached to the skull bone and soldered to a silver wire served as an electrical ground to the recording system. A three-axis accelerometer sensor attached to the headstage was used to record the X, Y, Z axes of movement of the head of the animals. Complementary, a 10-fold pre-amplification circuitry was located in association 4 cm distant from animals head, in order to reduce noise. The LFP signals were sampled at 1000 Hz, pre-amplified 500x and recorded in a Multichannel Acquisition Processor (MAP, Plexon System).

#### Open Field Apparatus

After one week of post-surgery recovery, animals were anesthetized with isoflurane (5%), for connecting the wires of the headstage with those of Plexon multi-electrode recording device. Around 5 minutes after that, animals were injected with saline (*Nacl*0.9%, 2 *ml*) and were placed in an open field apparatus (50 cm diameter and 30 cm high) at 10 am and recorded for 12 hours (Fig. 1(D)). During this time, animals were not disturbed in order to allow them to disclose a typical architecture of wake state, SWS, and REM sleep.

#### Identification of SWS episodes

Spectral analyzes of local field potentials were used to identify and quantify the occurrence of wake state, SWS, and REM sleep within the sleep-wake cycle (see Fig. 1(C)). State maps were employed for the characterization of waking and SWS/REM sleep states^30^, (ENeuFranca2015)^31^. We selected epochs of deep SWS within sleep clusters, located on the superior right border of the state map (see the shadow rectangle on the Fig. 1(C)). SWS states were behaviorally defined as presenting stillness with eyes closed at video recordings. Hippocampal LFPs obtained from deep sleep presented large-amplitude and slow-frequency oscillations and the head accelerometer signals were dominated by variations of very low amplitude. Only the first 4 recording hours were analyzed in this study. Sleep stages were grouped according to the following categories: WK (active exploration of the environment, large-amplitude variations on the accelerometer, and hippocampal theta (4–12 Hz) oscillations), SWS (stillness with eyes closed and large-amplitude slow (0.1–2 Hz) oscillations in the hippocampus) and REM sleep (stillness with prolonged whisking, no variations on the accelerometer signals, eyes closed and hippocampal theta oscillations).

#### Identification of micro-arousal with the accelerometer time series

Once we selected deep SWS episodes, we analyzed the occurrence of micro-arousal events on the accelerometer and LFPs signals [see Fig. 1(D) between dashed lines]^10^. Micro-arousal events were defined here as a fast motor-burst event during deep SWS on accelerometer signal with a minimum duration of 0.3 seconds, and an amplitude of more the two times the standard deviation of the basal signal (as previously described in^20,31^).

#### The *τ* (tau) event: the interval preceding the micro-arousal

The event *τ* interval was defined here as a duration of time beginning at the sharp drop in LPF amplitude at CA1 (>50%, using the Hilbert transformation envelope) [see Fig. (B) dashed line] and ends at the onset of the micro-arousal at Acc fluctuation (on average, the duration of *τ* is around 4 s). This pattern oscillatory CA1 LFP preceding motor explosions in Acc - during deep SWS - was persistently observed for all subjects (n = 176 in five animals). The statistic of the group for all 5 subjects who used ANOVA to test whether individuals present a distinct *τ* period did not find the significant difference (*F*(4,106) = 0.949, *p* = 0.44) [see Fig. 1 Supplementary Information].

#### Histological Preparations

In order to evaluate if electrodes were localized near the dorsal CA1 area of the hippocampus [see Fig. 1(B)], and at 1.5 mm deep in M1 and S1 cortical areas, brains were sliced and subjected to histological preparations. Briefly, after electrophysiological recordings, animals were euthanized in deep anesthesia with isoflurane (100%), and perfused with PBS (pH 7.4) and paraformaldehyde 4%; brains were removed, freezed at −80 °C and sliced at 30 um thickness. Nissl staining of coronal slices was performed in each brain of implanted animals, and in all cases brain tissular micro-lesions were located at 1.5 mm deep (cortex electrodes) or at CA1 hippocampal layer.

### Data Acquisition and Analysis

#### Data Treatment

**Figure 2:**
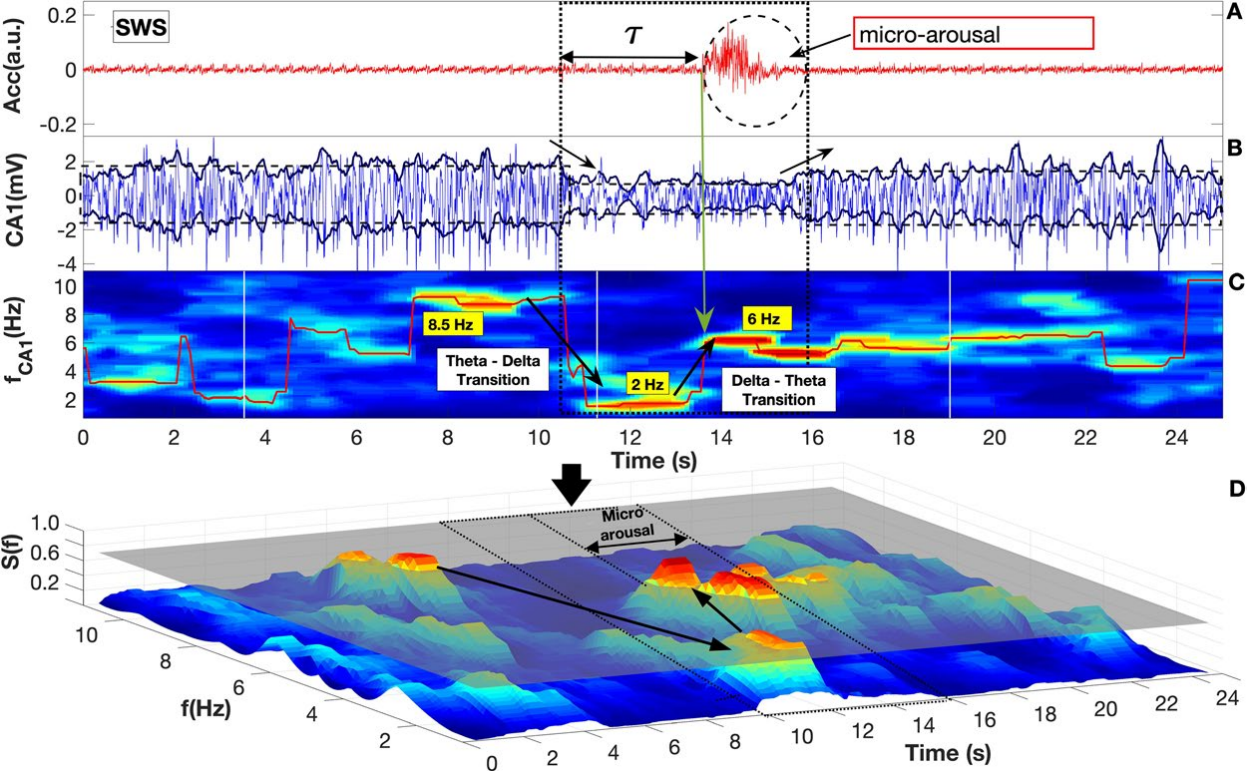
Hippocampus activity predicts micro-arousal in slow wave sleep. Panel (A) depicts the time series from Acc sensor placed on the animal head (arbitrary units). Panel (B) evidences CA1 Local Field Potential (LFP, mV), and the envelope using Hilbert transform of the signal (continuous dark blue lines). Horizontal dashed lines represent mean values of Hilbert transform for three epochs: before and during a *τ* time interval and after micro-arousal. Panel (C) displays the spectrogram obtained from (one example) CA1 data computed for a moving window of 1, 2 sec [see Method section]. To guide the eye we plotted a red line throughout the entire time showing the highest amplitude as a function of frequency and time. Panel (D) we display a three-dimensional spectrogram of CA1 signal shown in (**C**) normalized by the maximum amplitude of the entire spectrogram matrix. Over panels (A,B) vertical dotted lines indicate the beginning and the end of the changes in CA1 morphology dynamics (amplitude changes). Note a sharp drop of CA1 LFP amplitude during the *τ* plus micro-arousal intervals, highlighted by the envelope of the Hilbert transform. Panel (C) shows a first frequency transition from 8.5 Hz (high) to 2 Hz (low) (*θ* → *δ*) that characterize the beginning of the *τ* interval. A second frequency transition from 2 Hz to 6 Hz (*δ* → *θ*) occurs at the beginning of the micro-arousal (shown by the vertical up-side-down arrow full line). The normalized power spectrum involved in the frequency transition is shown in panel (D). A threshold plane located at 70% of the maximum power spectrum is depicted in order to show the bigger gap of energy involved in the frequency transition (*δ* → *θ*) compared to those occurring normally far from internal arousal.

The accelerometer output consists of three components of the acceleration vector *a*(*i*) = (*a*_*x*_, *a*_*y*_, *a*_*z*_). To perform the mathematical analysis we combined the vector components in a single quantity similar to the vector module at the same time that we extract the average of each vector component. The new *t*(*i*) vector formed from the components of *a*(*i*) is defined as; $Acc=({a}_{x}-{\hat{a}}_{x})+({a}_{y}-{\hat{a}}_{y})+({a}_{z}-{\hat{a}}_{z})$ where $\hat{a}$ is the mean of *a*, shown in Fig. 1 - the second panel in(D), and with more detail in Fig. 2(A)^20,31^.

#### Envelope analysis using Hilbert Transform

Knowing the analytic signal is a complex signal. Mathematically the envelope *e*(*t*) of a signal *x*(*t*) is defined as the magnitude of the analytic signal as: $e(t)=\sqrt{x{(t)}^{2}+\hat{x}{(t)}^{2}}$; the $\hat{x}{(t)}^{2}$ is the Hilbert transform of the *x*(*t*). For each LFP signals from CA1 Hippocampus area [Fig. 2(B)] and S1/M1 Cortex region we calculated the envelope using Hilbert transform and smothering it calculates the average of each 200 time window and shifting it using 1 dot as step.

#### Spectrogram Analysis

The Spectrogram (Fast Fourier Transformed Windowed) was calculated for 1.2 sec windows (1200 dots) and shifted every each 100 dots = steps (overlapping = 1,100 dots = 1.1 sec). The normalization of the spectrogram matrix was computed by summing all value of frequency’s amplitudes for each time column and dividing it for each corresponding frequency amplitude. Next step was filtering the spectrogram’s matrix to make it smoother: The procedure was taking each 20 dots (window) and calculate the average of it. After that we shifted the windows for every each 1 dot (19 dots of overlapping). To guide the eye about the highest amplitude as a function of frequency and time of the spectrogram matrix we computed the maximum of it of each time column and plotted the red dot on it. As result we have plotted a red line throughout the entire time as shown in Fig. 2 panels (B) and (C). Following the sampling theorem (Nyquist theorem) and once our frequency bands of interest are delta (0.5–4.0 Hz) and theta (5.0–8.0 Hz) we built our spectrogram matrix choosing the frequency interval from 0.5 Hz to 11.5 Hz with the accurate (step) of 0.1 Hz.

#### All Event-related Spectrogram Analysis

Spectrograms were calculated using the “spectrogram” function of MatLab’s Signal Processing Toolbox (2015b). We first obtained CA1, M1 and S1 LFPs signals 20 sec around all micro-arousal events across all animals. Then we computed spectrograms in 2-s Hamming windows with 1.5-sec overlap to LFPs from each of the three brain areas. We then normalized each spectrogram from each brain area using “zscore” function and calculated the mean across all spectrograms from all animals to obtain the averaged event-related spectrogram for each brain area.

#### Power Spectral Density

**Figure 3:**
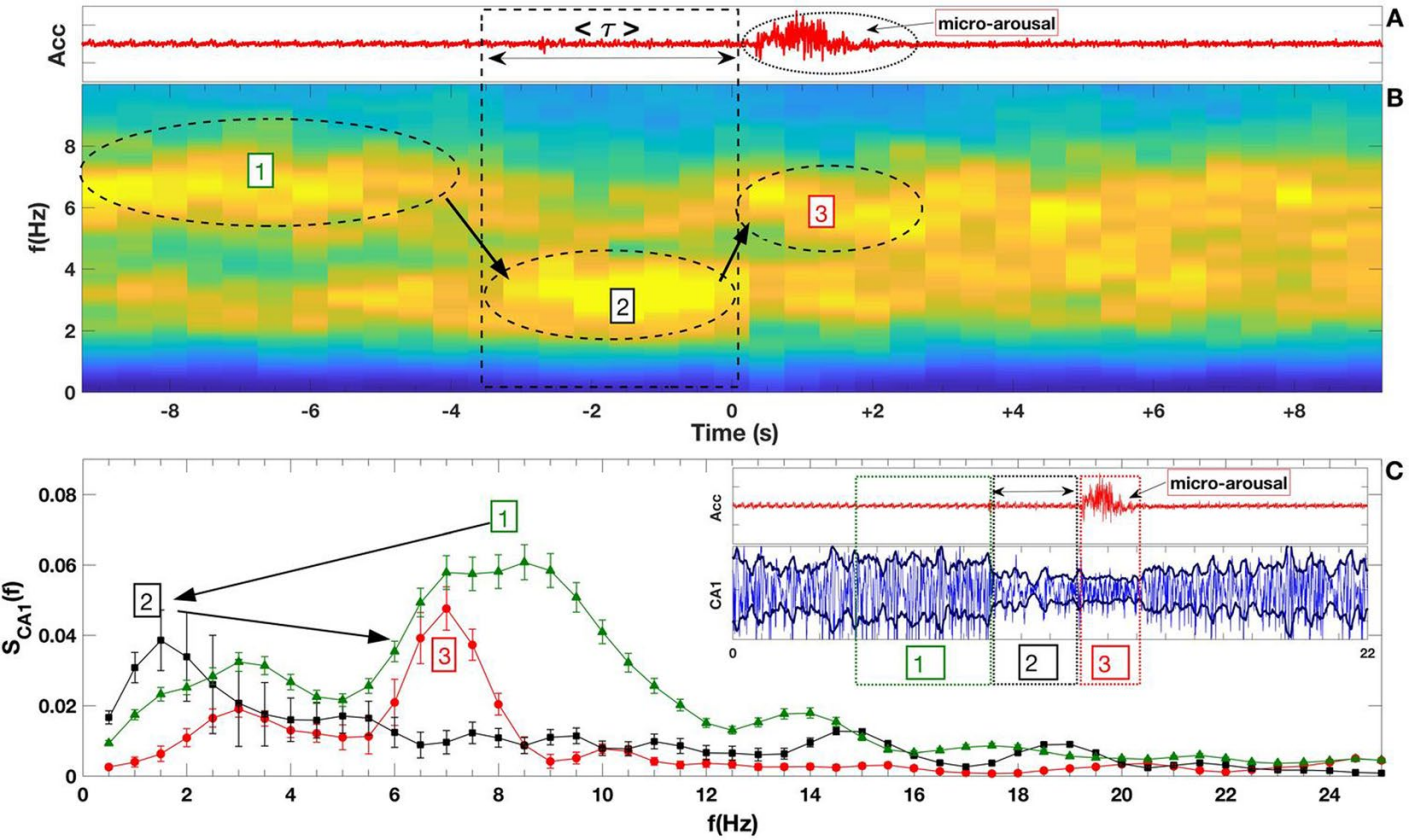
Hippocampal frequency transition around micro-arousal events. Panel (A) displays the accelerometer fluctuation (arbitrary units) for a typical event. Average of <*τ*> interval is shown by the double arrow. The oval dashed line highlights the micro arousal. Panel (B) shows normalized spectrogram averaged (n = 174 - five subjects) obtained from slow wave sleep stage with micro-arousal. The oval dashed lines depict the dominant frequencies amplitude throughout the three epochs: [1] indicates the interval preceding the *τ* interval; [2] shows the *τ* interval and [3] indicates the micro-arousal events. The arrows show the transition between the dominant frequencies as a function of time. Panel (C) display the mean power spectrum from the three-time intervals with the transition between the dominant frequencies (arrows). The bars in the power spectra depict the standard error. The inset panel shows a standard example of micro-arousal dynamics: ([1], green) interval of time preceding the *τ* interval, ([2], black) period of time corresponding to the *τ* interval, and ([3], red) time interval corresponding to the micro-arousal events.

We calculated the Power Spectral Density from CA1 Hippocampus and S1/M1 Cortex signals of all 5 mice of all deep sleep episodes during three epochs: [1] during the short time preceding *τ* interval; [2] during the *τ* interval; [3] during the micro-arousal interval as shown in Fig. 3 panel (C). The average (mean) and standard deviation of the mean for all event were calculated and plotted.

#### Event-related Coherogram Analysis

**Figure 4:**
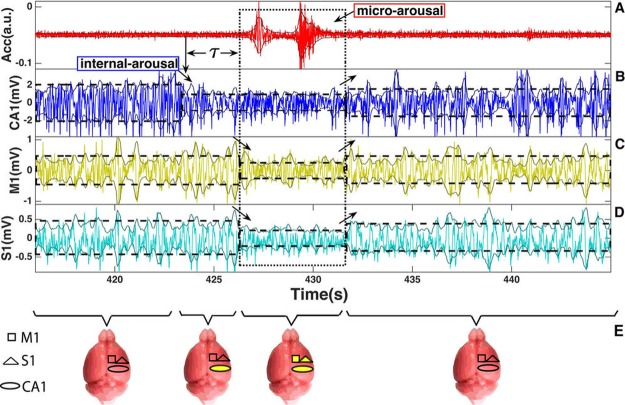
Hippocampus and Cortex LFP dynamics associated with micro arousal response. Panel (A) shows the raw accelerometer (Acc) signal, in arbitrary units (a.u.), while lines (**B**–**D**) indicate the hippocampal local field potentials (in mV) of CA1 area, primary motor area (M1) and primary somatosensorial area (S1), respectively; line (**E**) illustrates the spacial-temporal brain dynamics in micro-arousals: CA1, S1, and M1 presents initial (leaked geometric figures) and changed (full yellow figures) dynamic patterns in brain areas, which are illustrated by squares (M1), triangles (S1) and ellipses (CA1). The envelope lines around CA1, M1, and S1 are the output from Hilbert transform, which was used to analyze changes in signal dynamic. The CA1 area exhibits a change in dynamics (an intrinsic micro-arousal) around 3 seconds (*τ*) before the start of muscle high amplitude activity in Acc. During the micro arousal motor activity period (highlighted by a dotted line in all graphs), also M1 and S1 areas exhibit a change in signal dynamics. This change in LFP dynamics is characterized by a reduction in average amplitude, firstly in the hippocampus and followed by the cortex, as indicated by dashed lines and arrows in each case. Notice that after the micro-arousal motor activity interval all signal patterns from accelerometer and brain LFP amplitudes return to the initial state.

**Figure 5:**
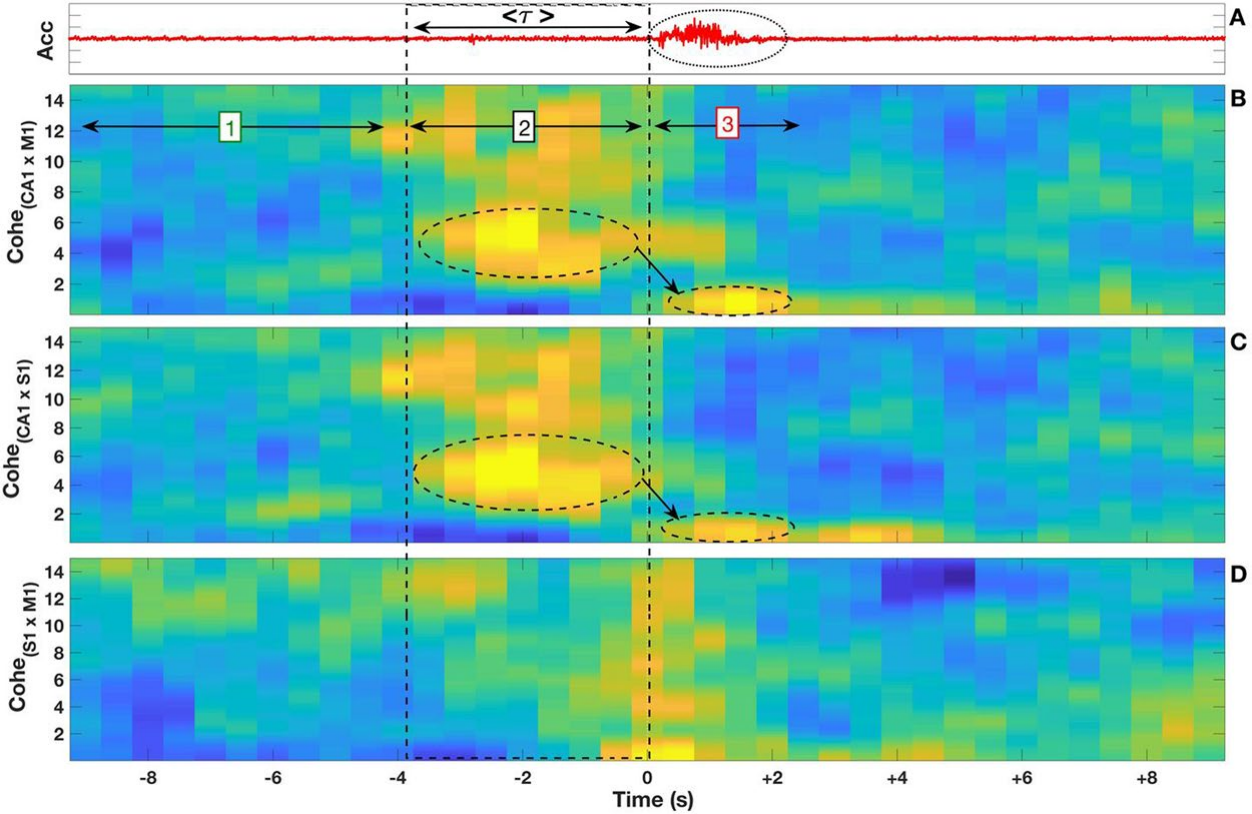
Coherence between cortical and hippocampal areas around the micro-arousal events. In panel (A) the accelerometer signal is shown, the arrow depicts the *τ* interval. The oval dashed lines highlighted the micro-arousal event. Panel (B) display the normalized coherogram between CA1 and M1. The oval dashed line highlighted the peak coherence frequency. Arrows depict [1] time intervals preceding *τ* [2], the *τ* interval, and [3] the interval corresponding to the micro-arousal event itself. Panel (C) shows the normalized coherogram between CA1 and S1. These two cases, involving the hippocampus, show a peak coherence during the *τ* interval (preceding micro-arousal) in the theta frequency band. Panel (D) shows the normalized coherogram between S1 and M1. In this case, the coherence appears at the beginning of the micro-arousal. The dashed square mark along the panels highlight the *τ* interval preceding the micro-arousal. The coherograms in this figure show an average statistics over all events.

**Figure 6:**
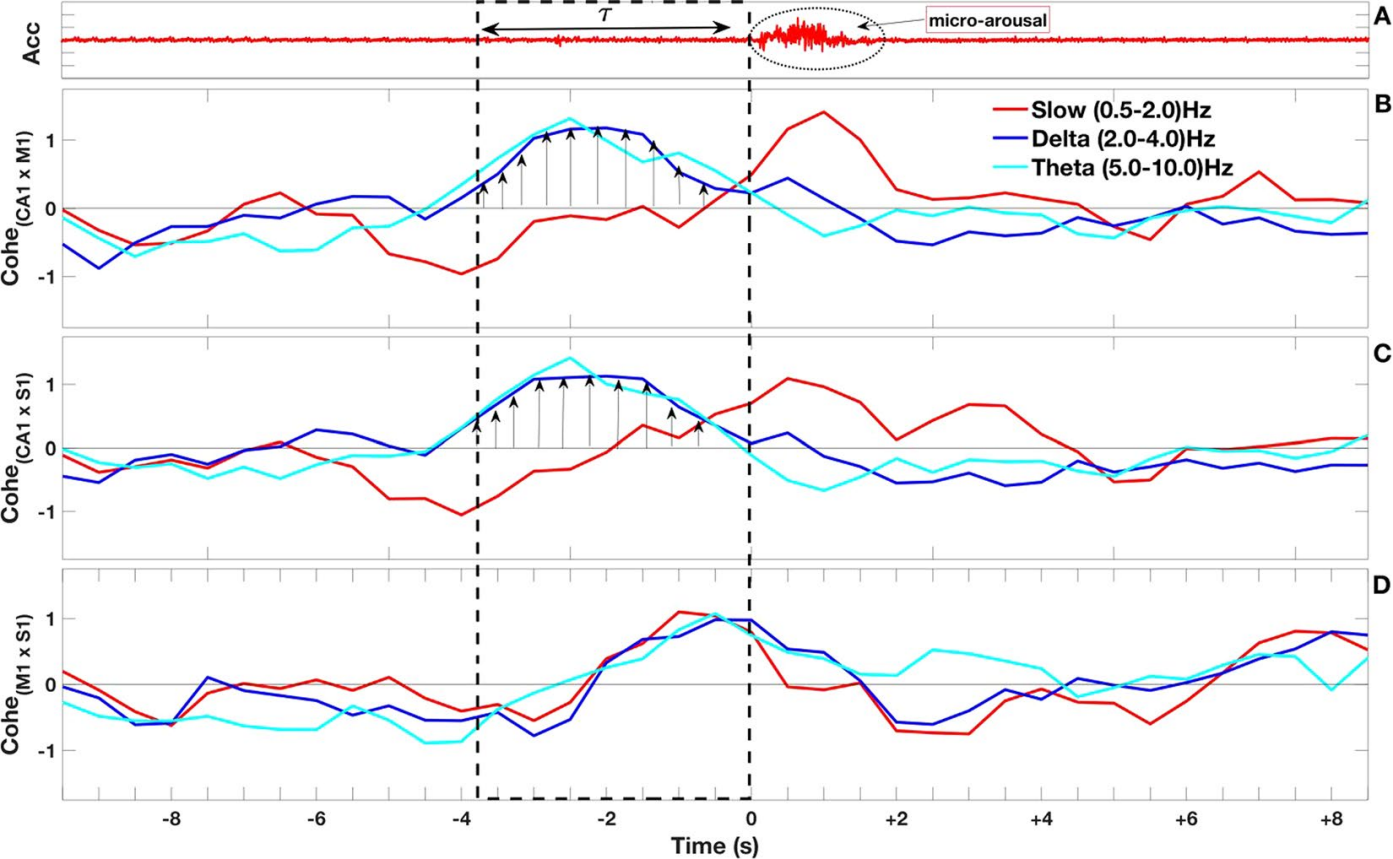
Coherence between cortical and hippocampal areas focusing on brain oscillations. We separately analyze the following band frequencies: slow (0.5–2 Hz), delta (2–4 Hz), and theta (5–10 Hz) for a 10 s interval around micro-arousal events. (**A**) Accelerometer signals display a typical micro-arousal event during slow-wave sleep, as before we indicate the *τ* interval and the micro-arousal. In the following panels, we plot the averaged normalized coherence at slow, delta, theta frequencies between CA1 and M1 (**B**), between CA1 and S1 (**C**), and between S1 and M1 (**D**). The dashed rectangle highlights the *τ* interval. The arrows in panels (B,C) indicate a high coherence between hippocampal and cortical areas during the *τ* interval. We remark also a peak coherence in the cortex at the onset of the arousal.

The coherence function is a frequency domain measure of the similarity of two signals x(t) and y(t). The magnitude-squared coherence function (MSC), which is a function of frequency, is given by formula: ${C}_{xy}(f)=\frac{|{S}_{xy}(f){|}^{2}}{{S}_{xx}(f){S}_{yy}(f)}$. When *S*_*xy*_(*f* ) is the cross-spectrum density between the x and y signals, and *S*_*x*_(*f* ) or *S*_*y*_(*f* ) are the individual power spectra of signals x and y respectively. The coherence of our physiological signals was calculated using the “mscohere” function (see above) of Matlab’s Signal Processing Toolbox (2015b). We first obtained CA1, M1 and S1 LFPs signals 20 s around all micro-arousal events across all animals [see Fig. 4]. Then we computed the coherence in 1-s Hamming windows with 750-ms overlap between pairs of LFPs from all the three brain areas as displayed in Figs 5 and 6. We then normalized each coherogram using zscore function (that returns the z-score for each element of a specific signal such that columns of this signal are centered to have mean 0 and scaled to have standard deviation 1) and calculated the mean across all coherograms from all animals to obtain the averaged event-related coherogram.

#### Statistical Analysis of the Mean Coherence across micro-arousal events

**Figure 7:**
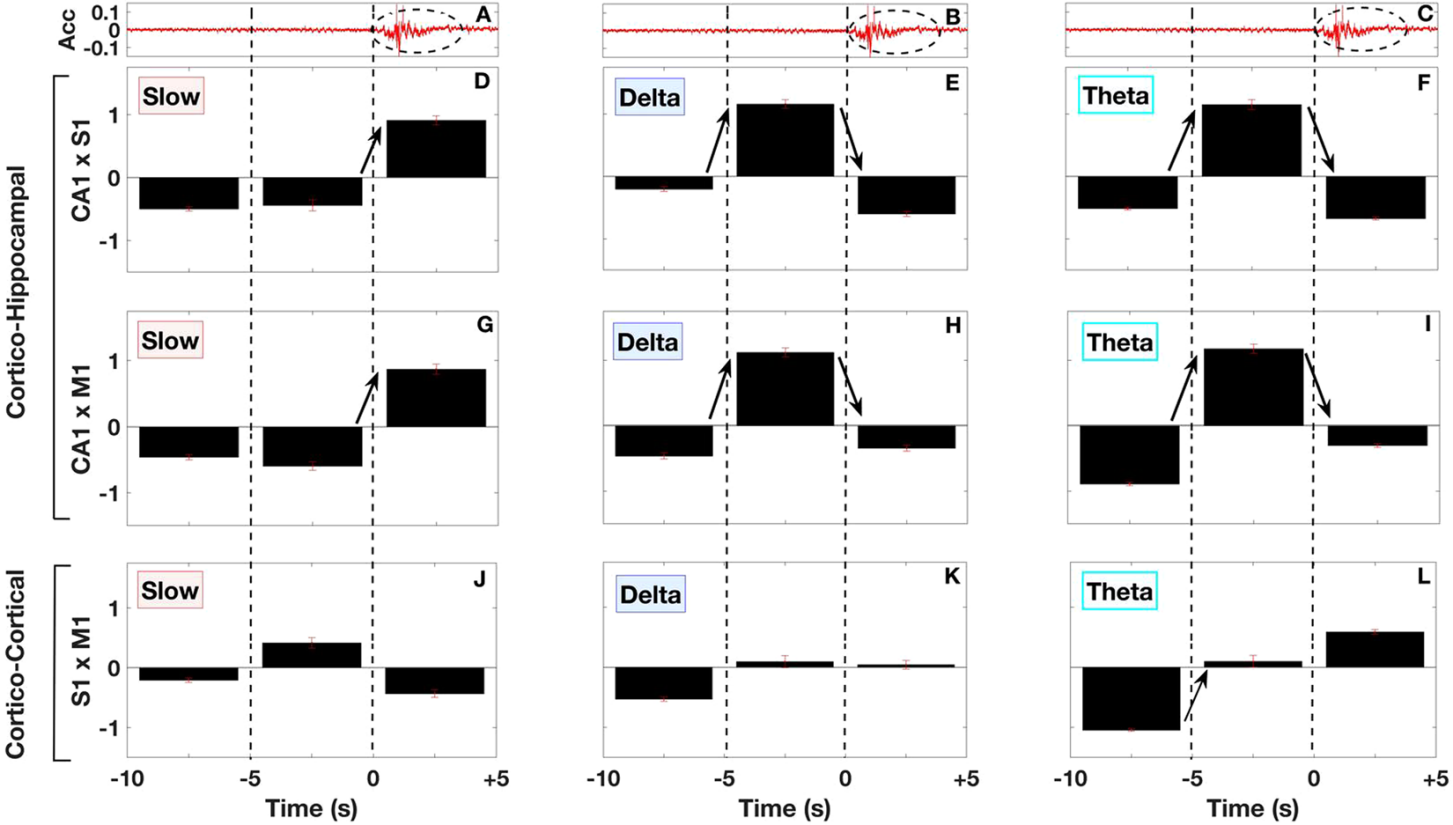
Group average coherence statistics between brain regions associated with frequency bands around the micro-arousal during SWS. Figure display the mean and standard error medium of coherence between hippocampal and cortical brain areas in intervals classified by frequency band and according to 5 second time intervals, depict in black blocks (in average pre-*τ*: −10s to −5s; in average *τ*: −5s to 0 s and post-*τ*: 0 s to +5 s) around the micro-arousal events. Panels (A–C) display coherence between CA1 and S1; Panels (D–F) depicts coherence between CA1 and M1 and Panels in the bottom line (G–I) present coherence between S1 and M1. In the three columns, we specified Slow (left), Delta (middle), and Theta (right) frequency bands. The vertical dashed lines in all panels indicated the beginning of the micro-arousal event (time zero). All significances at Tukey post-hoc test (after performing ANOVA comparisons) were indicated by bars and asterisks in order to guide the reader for the statistic differences related to changes of coherence. In Delta and Theta frequency band scenario, we observe a high amplitude positive value at the center position in (**B**,**C**,**E**,**F**) revealing an increased coherence between CA1 and M1/S1 during the *τ* interval. On the other hand, the Slow frequency band shows a positive high value just after the *τ* interval, changing from anti-coherence to high coherence at the onset micro-arousal event. In the cortico-cortical coherence (bottom line) ANOVA revealed only in Theta band a significant coherence change of amplitude from pre-*τ* to *τ* time blocks.

For comparisons of cross-structural LFP coherence among blocks of 5 s time intervals around micro-arousal events, we obtained the average normalized (zscored) coherence from all micro-arousal events (n = 167). We analyzed three bands of interest: Slow oscillations (0.5–2 Hz), Delta oscillations (2–4 Hz), and Theta oscillations (5–12 Hz) as shown in Fig. 7. We used one-way analysis of variance (ANOVA) followed by Tukey multiple comparison test, to find significant (*p* < 0.05) variations in the cross-structural LFP coherence across time (at pre-*τ*, *τ* and post-*τ* intervals) for each of these bands of interest. Data are expressed as mean ± SEM.

## Results

We studied hippocampal and cortical LFPs simultaneously recorded with head acceleration of 5 freely moving mice along the SWS stage (see Fig. 1). In this work we performed our analysis at periods of deep SWS presenting short bursts of motor-activity, which characterize micro-arousals as observed in Fig. 1(D). Moreover, we explored in detail the dynamic of LFP activity preceding micro-arousals across brain areas. Further, using coherence between hippocampal and cortical rhythms we detected a specific frequency-band communication preceding micro-arousal during SWS.

### Hippocampal dynamics organization preceding SWS micro-arousals

Our analysis revealed a multitude of motor-bursts on Acc activity (micro-arousals) that persistently occurred even during deep SWS stages. These micro-arousal events were consistently associated with periods of a significant drop in the CA1 LFP amplitude [see red rectangles in Fig. 1(D)], resembling patterns of brain activity typically observed during awake states. Panels (A) and (B) in Fig. 2 show examples of Acc and CA1 LFP activities around micro-arousal. Using Hilbert transform envelop, we observed a sharp drop in the CA1 LFP amplitude, which occurs a few seconds before the micro-arousal that we defined here as *τ* interval (2.2 seconds in this example). This CA1 LFP oscillatory pattern preceding motor-bursts in Acc during deep SWS was persistently observed for all individuals (n = 176 in five animals). In order to further investigate this LFP dynamics pattern around micro-arousals, we also analyzed the brain fluctuation frequencies over time. Figure 2(C), displays a spectrogram obtained from CA1 LFP. We firstly observed the rising of theta oscillations a few seconds before the onset of *τ*, followed by a frequency-band transition from 8.5 Hz to 2 Hz at *τ* onset (theta-delta). Hippocampal delta oscillations (2 Hz) remain stable throughout the *τ* interval. A second transition from 2 Hz to 6 Hz (delta-theta) occurs now precisely at the beginning of the micro-arousal. Please note that the CA1 low amplitude (starting at the *τ* onset) lasts until the end of the micro-arousal event. The CA1 LFP amplitude increases again, after the micro-arousal event. Further, in order to highlight the transition of dominant frequencies, in panel (D) we display a three-dimensional spectrogram of the CA1 signal shown in (C), and normalized by the maximum amplitude of the entire spectrogram matrix. A threshold at 70% of the maximum power spectrum was used to quantify the rate of frequency transition. We observed that 83% of all micro-arousal events were above the threshold for the two frequency-band transitions (theta-delta-theta). More examples can be found in Supplementary Material.

To confirm whether frequency jumps emerge in the group average, we performed the mean spectrogram and power-spectrum analysis around micro-arousal events (*n* = 174). Figure 3(A) depicts the Acc signal with a typical micro-arousal event, in which the average *τ* interval is 3.6 seconds [see Supplementary Information in Fig. 1]. Panel (B) shows the normalized mean spectrogram for a fixed time window (20 seconds) around the beginning of all micro-arousal events. The same outcome observed in Fig. 2(C) can be seen in the group results: a first frequency band transition (theta-delta) occurring at the onset of the average *τ* interval; and a second frequency band transition (delta-theta) occurring at the micro-arousal onset. Although the analysis here overlaps all events with different *τ* duration [see Supplementary Information], we observe a dominant power-spectrum for different intervals of time: (i) theta oscillations at pre-*τ* interval from −10 to −4 s; (ii) delta oscillations at the average *τ* interval itself, and (iii) a lower theta oscillations after *τ* interval (in average) that includes the micro-arousal/motor-burst activity itself (panel (A) related to Acc data).

To further investigate the precise frequencies that characterize each interval around micro-arousal events, we performed Fourier transform for three specific intervals: [1] pre-*τ* [2], *τ*, and [3] post-*τ* (see Fig. 3(C) inset and also Fig. 2(A,B)). Figure 3(C) shows the mean power spectrum of these three defined intervals for all micro-arousal events. We observe two main frequency bands in the pre-*τ* interval [1] (green): one between 5–12 Hz (maximum at 8.5 Hz) and the other as a lower peak whose local maximum is around 3 Hz. In its turn, the *τ* interval itself [2] (black) disclose only a slower oscillation (0.5–4.0 Hz, peak at 1.7 Hz), which corresponds to classical delta band. Finally, at post-*τ* interval [3] (red) we verified a sharp frequency range of 6–8 Hz, which peaked around 7 Hz. Noteworthy, pre-*τ* and post-*τ* intervals exhibit theta oscillations with different frequencies. While pre-*τ* is dominated by a wide theta frequency band (encompassing both type-I related to atropine resistant theta: 8–14 Hz; and type-II related to atropine sensitive theta: 4–8 Hz), the post-*τ* interval is related specifically to theta type-II^32–35^. Taken together, these results show a unique dynamic behavior in the hippocampal LFPs that rises seconds before micro-arousal events. Aiming to investigate sensory and motor cortical processing linked to micro-arousals during SWS, we next analyzed cortico-hippocampal communication around motor-bursts.

### Hippocampal and cortical frequency-band communication preceding micro-arousal

In order to investigate sensory and motor cortical processing linked to micro-arousals during SWS, we next analyzed cortical and hippocampal dynamics around motor-bursts. Figure 4 shows simultaneously recorded Acc signals (panel A), hippocampal (CA1, panel B), primary motor (M1, panel C) and primary somatosensory (S1, panel D) LFPs around a typical micro-arousal event. The sharp drop in hippocampal CA1 LFP amplitude observed during *τ* interval (indicated by *τ* in Fig. 4(B)) is not simultaneously accompanied by cortical M1 and S1 amplitude transition. The abrupt drop in the amplitude of S1 and M1 cortical LFPs often occurs simultaneously at the micro-arousal onset (see arrows in Fig. 4 panels C and D). This example represents the average LFPs amplitude and Acc signals around micro-arousals in the whole group of mice. Other examples of amplitude transitions can be verified at Supplementary Material at Fig. 2. In addition, using the same approach as Fig. 3(C), we performed Fourier transform from M1 and S1 cortical LFPs to investigate in more detail frequency ranges at intervals: pre-*τ*, *τ*, and post-*τ*. Differently, from hippocampal LFP that oscillates from 0.5 to 12 Hz, cortical LFPs oscillated basically only beneath 4.5 Hz, which corresponds to delta and slow rhythms [more details see Supplementary Information]. Specifically, we found a subtle peak frequency difference between M1 and S1 during the *τ* interval: while in S1 the peak is located around 2 Hz, the M1 peak is situated around 3.5 Hz [see Supplementary Material]. To further describe these temporal changes in multiple cerebral LFP fluctuations, we model four main intervals: pre-*τ*, *τ*, post-*τ*, and return to basal, where ellipses mean CA1 region, squares mean M1 region and triangles mean S1 region (Fig. 4(E)) and color represents changes in amplitude fluctuation characteristics.

Since previous works demonstrated cortico-hippocampal anatomical communication^27^ and once our results revealed that amplitude transitions in CA1 precedes those in M1 and S1, we performed frequency coherence analysis to evaluate a possible cross-talk among those areas around micro-arousal events. Figure 5 shows the coherence between cortical and hippocampal areas ±10 sec. around the micro-arousal events. Panel (A) shows the Acc, evidencing *τ* (in average) and the onset of the micro-arousal event (oval dashed line). Panels (B–D), display, respectively, the normalized coherogram between brain areas: CA1 × M1; CA1 × S1; and M1 × S1, in average overall micro-arousal events. In Fig. 5 we observed no prominent frequency coherence between brain areas during pre-*τ* [1] interval. We observed an increase of cortico-hippocampal coherences [panels (B) and (C)] that match exactly *τ* interval duration [2], which emerges in delta and theta frequency bands and peaks (2.5–5.5 Hz)(dashed ellipses). We also noted a remarkable high coherence transition from 2–6 Hz to 0.5–2 Hz frequency bands that occurs specifically at the onset of micro-arousal (post-*τ* interval [3]) [see oblique arrows in Fig. 5(B,C)]. Conversely, cortico-cortical coherence [panel D] only rises around micro-arousal onset, in a wide range of frequencies (0.5 Hz to 12 Hz).

To better investigate the coherence around micro-arousal [shown in Fig. 5], we measured the three frequency bands of interest as time function: Slow oscillation (0.5–2 Hz, in red), Delta range (2–4 Hz, in blue) and Theta range (5–10 Hz, in cyan), as observed in Fig. 6. Panel (A) displays accelerometer signal (Acc). The normalized coherences between CA1 × M1 are shown in panel (B), CA1 × S1 in panel (C) and S1 × M1 in panel (D). We verified: (i) no prominent coherence between cortico-hippocampal as well as cortico-cortical LFPs during the pre-*τ* interval (around −10 to −5 sec.) for all frequency bands at panels (B–D) [see Fig. 6]. (ii) a marked increase of the coherence between all brain areas during *τ* interval (in average from −4 to 0 sec.). However, in a closer view, Delta (blue) and theta (cyan) cortico-hippocampal coherences increase at *τ* onset and reaches its maximum positive value around half of the *τ* interval (about −2 sec.). On the other hand, slow oscillations (red) present slightly different temporal increase: at *τ* onset it reveals a maximum negative value and slowly gain positive values across *τ* interval, reaching its maximum positive value only at post-*τ* interval [see set of arrows in panels (B) and (C)]. Panel (D) shows that cortico-cortical coherence in these three frequency bands increase simultaneously during *τ* interval and reach its maximum values near the transition from *τ* to post-*τ* (around −1 to 0 sec.). (iii) that cortico-hippocampal coherences for slow oscillations (red) reveals its maximum positive values during post-*τ* interval (0 to 5 sec.). Conversely, coherences of delta and theta bands decrease returning to basal levels after micro-arousal [panels (B) and (C)]. Cortico-cortical coherence [panel (D)] at post-*τ* interval reveals a progressive decrease for all three analyzed band frequencies. Also, a slight coherence difference between delta and theta range rhythms can be perceived in detail: while slow and delta oscillations fall to negative values after *τ*, theta oscillation presents a faint fall but remains at positive values [see Fig. 6(D)].

Additionally, we checked whether frequency communication between brain areas displays statistical differences among pre-*τ* (from −10s to −5s), *τ* (−5s to 0 s) and post-*τ* (0 s to +5 s) intervals performing ANOVA comparisons (see Methods). Figure 7 depicts cortico-hippocampal and cortico-cortical coherences in boxes with 5 a seconds duration each. For all panels, the micro-arousal onset was used as reference (zero second).

The analysis of cortico-hippocampal frequency communication confirmed that coherence in Slow oscillations (panels A and D) increases significantly during the post-*τ* interval (CA1×S1: p = 0.002, F = 7.74, and CA1×M1: p < 0.001, F = 9.37; Tukey post-hoc p < 0.05, considering pre-*τ* and post-*τ* intervals in A and D). Also, statistical analysis of coherence in Delta revealed important significant differences (CA1×S1: p < 0.001, F = 15.51 and CA1 × M1: p < 0.001, F = 9.73; Tukey post hoc revealed in both cases an increase at *τ* interval, p < 0.05 compared to pre-*τ* and post -*τ* intervals). The analysis of coherence at Theta band considering cortico-hippocampal communication worked in the same direction of previous results at Delta, showing significant differences (HPxS1: p < 0.001, F = 17.13; HPxM1: p < 0.001, F = 15.67; Tukey post-hoc revealed in both cases an increase at *τ* interval, p < 0.05 compared to pre-*τ* and post -*τ* intervals) [see asterisks in panels (B, C, E, F)]. On the other hand, the analysis of cortico-cortical coherence shows no differences in slow (S1 × M1: p = 0.09, F = 2.5) or delta (S1 × M1: p = 0.49, F = 0.73) bands [see panels (G, H)], but theta (S1 × M1: p < 0.001, F = 9.82) band presents a significant increase from the pre-*τ* to *τ* intervals (p < 0.05 at post-hoc Tukey test) [see panel (I)]. Therefore, the foremost results here show that while cortico-hippocampal communication in delta and theta bands increase at *τ* onset [(panels B, C, E, F)], while cortico-cortical coherence presented only marginal differences (maximum p-value of 0.09, related to panel G) around the micro-arousal events. For all F values the degrees of freedom were (2, 29). In summary, here we described a distinct dynamic pattern around micro-arousals in the mice brain, and these results point to an activity shift occurring firstly at the hippocampus (CA1) and, in sequence, at primary cortical areas (S1 and M1).

## Discussion

According to the American Sleep Disorders Association definitions, physiological arousals could be considered events of sleep disruption, thus potentially signifying a risk of breaking a regular sleep pattern. However, recent studies pointed out that micro-arousals can be considered as an intrinsic part of sleep architecture, having a physiological role in its regulation and maintenance^9,10,36–38^. Results evidenced here corroborate those present-day views, in which micro-arousal events may be critical for the proper maintenance of SWS sleep state and disclose a complex but robust structure pattern. This idea is supported by our results, which gives the interpretation of neural processes providing a preparation (*τ* interval) for the emergence of motor-bursts (micro-arousal) in sub-cortical structures (such as the hippocampus). In detail, these processes are associated with LFP changes in frequency and amplitude parameters during SWS sleep. Furthermore, the increase of cortico-hippocampal coherence precedes the increase of this parameter in cortico-cortical networks [see Fig. 6]; these outcomes point out to the same direction of the previously described ascending reticular activating system (ARAS). In this sense, our coherence results indicate that cortical and motor activation are the culmination of a gradual processes, during the *τ* interval, performed to promote the rise of micro-arousals.

Other events in *τ* interval seem to be critical for the micro-arousal emergence. In the past related work, using the same acquisition methods, Lima *et al*.^20^ described that the accelerometer signal discloses a typical decrease in Determinism (DET, an output of recurrence plot analysis) before SWS micro-arousals, thus serving as an arousal predictor. The decrease in DET starts approximately 4 seconds before the micro-arousal onset, comprising an interval which coincides with *τ* interval as here described. This implies that, at *τ* interval, motor activity displays a gradual loss of entropy which coincides in time with a progressive increase in cortico-hippocampal frequency coherence. Also, as reported by Lima *et al*.^20^, around 4 seconds preceding micro-arousals, amplitude fluctuation, in the hippocampus, start to decrease, which coincides with the amplitude drop onset in this present paper. All these data also support the idea of a well-defined and gradual process occurring in sub-cortical, cortical and motor structures in the transition interval (*τ*) allowing micro-arousal emergence. However, these data do not yet really explain why micro-arousals emerge in SWS state, in terms of an adaptive physiological mechanisms.

So, from the descriptions evidenced here, we built two rationales to possibly situate micro-arousals as crucial events within SWS physiology. One, the micro-arousal may work as a regulatory process in deep sleep homeostasis, probably to avoid an excessive deepening of sleep that could lead to a pre-comatose sleep stage. The fact that both hippocampal and cortical LFP and accelerometer signal returns to basal states after motor-bursts provides some support for this consideration. In the second rationale, the micro-arousal may emerge as a result of an incomplete SWS transition for REM sleep. This last hypothesis is supported in our work by the occurrence of theta before and after a brief period of slow oscillation in CA1. Finally, these two rationales are not mutually excluding: a mouse could avoid an excessive deepening of SWS state through an incomplete (a micro-arousal with a return to the basal state) or even a complete transition to the REM stage.

Past works defined as SIA a small irregular activity that appears in hippocampus in varied phases of sleep/wake cycle^17^. Here, however, we specifically selected similar events related to SIA definition that were observed within SWS episodes. This was performed because recently, hippocampal LFP amplitude changes during SWS were associated with micro-arousal episodes in sleep^20^. In addition, theta rhythm was associated with the onset of the micro-arousal event revealed by accelerometer data (Acc), following similar results presented by Kang *et al*.^39^. Theta oscillations can be found in two sub-band frequencies described for hippocampal LFP: type-1, related to atropine-resistant (8–12 Hz) and type-2, related to atropine-sensitive (4–8 Hz)^33,34^. Our findings point to the occurrence of both high (8–12 Hz) and low theta (4–8 Hz) before shifting to slow oscillation/delta range (first frequency jump) and in sequence from slow oscillation to a lower theta range/type-2 theta (second frequency jump) [see Figs 2 and 3].

In detail, we describe frequency band transition associated with micro-arousals in a series of three main time intervals in both hippocampus and cortex: (i) Before micro-arousals (pre-*τ* interval), hippocampal oscillations are situated in both bands of type-1, atropine-resistant theta and in type-2 theta (atropine sensitive). (ii) In sequence, *τ* interval, there is a CA1 frequency band transition (jump) from theta to delta band (around 2.0 Hz), while in S1/M1, delta arises within a (1.5–4.0 Hz) band [see Supplementary Information Fig. 4]. (iii) Micro-arousal begins with the onset of a type-2 CA1 theta range (around 6.5 Hz). Such band was not found in the cortex in a high power-spectrum in this post-*τ* interval [see Supplementary Information Fig. 4]. However, we observe a cortical LFP amplitude decrease by the micro-arousal onset. Previous work described the existence of type-1 and type-2 theta bands during SWS sleep at the onset of arousals^39^ but, at wake state, type-2 theta is considered extremely fugacious. Type-1 theta (8–12 Hz) is associated with locomotion and some voluntary behavioral patterns, and, complementary, it is found in REM sleep, while type-2 theta (4–7 Hz) is typically a transient, short living rhythm, encountered when an animal is preparing to move or when it is frightened in a freezing state^32^. Conversely, our findings point to a different function of both theta types in SWS stage: around micro-arousals, type-1 theta appears when the animal does not present relevant motor activity (quiet behavior), while type-2 theta emerges when it performs a motor-burst. In light of the present data, we can not point to precise mechanisms related to this finding, and more research would be useful in this field. Nevertheless, because both theta-2 from REM and from micro-arousals may be related to the emergence of superficial sleep phases, they may arise through shared neurotransmitter networks, such as the cholinergic input from septal area^40^.

Concerning slow oscillation appearance in CA1, we detected delta-related frequencies immediately before micro-arousals. There are two types of slow rhythms in the hippocampus (0.5–4.5 Hz). One is the hippocampal slow oscillation, coupled to cortical LFPs, with a peak frequency of 0.64 ± 0.05 Hz (described in urethane-anesthetized rats)^41^. The other is the hippocampal respiration-induced rhythm (HRR), peak in 1.25 ± 0.10 Hz (described in urethane-anesthetized rats), and around 3.5 Hz (in mice)^41,42^, whose detection in hippocampus is dependent of airflow through mouse nostrils and the consequent respiratory rhythm generated in the olfactory bulb. This information enters in hippocampal networks through the entorhinal cortex^42^, which is also entrained by olfactory bulb respiratory pattern. According to Yanovsky and co-authors^42^, mouse respiration rate under urethane anesthesia (2.0–4.0 Hz) fulfills the range of HRR oscillations, but this rate may be different in a deep SWS physiological state. Usually, both brain-related olfactory bulb rhythm and respiration rate in mice appears in non-REM sleep stages (1.2–3.5 Hz), when detected with electrodes and simultaneous plethysmography^43^. This rate is consistent with our findings, which reveals that mice, in this work, disclose in CA1 peaks in the delta (1.0–3.5 Hz), which may reflect a respiratory pattern [see Fig. 3(C) - black circle/line during *τ* interval].

Taking a closer look at the results concerning frequency coherence analysis, the range of slow oscillations band (1.5–3.5 Hz), potentially associated with HRR, have a specific profile in cortico—hippocampal coherence, which is markedly different from the hippocampal slow oscillation (0.5–2 Hz): before micro-arousals, we have an increase of coherence of HRR-compatible oscillations and theta bands between hippocampus and cortex; immediately after the arousal this coherence is diminished. On the other hand, cortico-cortical coherence in all frequency bands increases during a micro-arousal event or until 2 seconds after this time point [see Fig. 5(D)]. From this observation, we may argue that, immediately before the micro-arousal, coherence between somatomotor cortex and sub-cortical structures (such as the hippocampus) in both HRR and theta rhythm, may be an important phenomenon linked to the onset of the arousal event. However, more studies are necessary to indicate a clear causal relationship between the increase of cortico-hippocampal communication related to these frequency ranges, in *τ* interval. These studies may include the analysis of coherence variance regarding respiratory-derived rhythms, associated with the micro-arousal event in SWS architecture. Considering that micro-arousals might appear as a result of oxygen and carbonic acid body fluctuations in SWS, future works also should focus on olfactory bulb patterns, associated with respiratory markers, O2, and C02 blood levels, and to hippocampal LFP oscillations, within the context of micro-arousals, in order to clarify this possible mechanism.

Additionally, concerning cortico-hippocampal communication, a previous study showed a robust correlation of neuronal discharges between the somatosensory cortex and hippocampus in different time scales in the mouse and rat^27^. Considering global neuro-anatomical and neurophysiological basis of arousal processes, we may interpret our results in terms of an increase in frequency-based communication considering hippocampus and cortical areas before the arousal onset. This makes sense, since hippocampus may work together with other sub-cortical structures, such as the thalamus in the ascending activating system, to activate the cortex and prepare for a possible arousal or another transitional state. Our results also suggest that, after the micro-arousal onset, hippocampal CA1 area decreases the coherence with the somatomotor cortical areas, while cortico-cortical communications between sensory and motor primary areas gradually become more relevant to process information required considering the imminence of a possible return to the waking state.

As a concluding remark, we described here a unique dynamical pattern of hippocampal and cortical activation associated with micro-arousals during sleep. Future questions raised by these descriptions could be addressed by a future similar detailed analysis comprising cortical, hippocampal and accelerometer signals, of SWS transition events to REM stage, in order to test if micro-arousal emergence in SWS shares common dynamical features with SWS-REM transitions. Complementary, the use of agonists and antagonists of sleep-related neurotransmitter pathways, such as acetylcholine, dopamine, and serotonin, could better illustrate their critical role in the onset of micro-arousals. The importance of these findings is related to research on SWS dynamical role and regulation, as well as to clinical studies involving pathological arousals associated with sleep disorders.

## Supplementary information

Supplementary Information
