# Supplementary material for: Hippocampal and cortical communication around micro-arousals in slow-wave sleep
Source: Sci Rep. 2019 Apr 10;9:5876. doi: 10.1038/s41598-019-42100-5 (PMC6458146; doi:10.1038/s41598-019-42100-5)
Supplement: Supplementary file 1 — Supplementary Information [file 41598_2019_42100_MOESM1_ESM.pdf]

Supplementary Information

"Hippocampal and cortical communication around micro-arousals in slow-wave sleep".

Authors:

Dr. Gustavo Zampier dos Santos Lima,
Dr. Bruno Lobão-Soares,
Dr. Gilberto Corso,
Dr. Hindiael Belchior,
Dr. Sergio Roberto Lopes,
Dr. Thiago Prado,
Dr. George Nascimento,
Dr. Arthur Cavalcanti de França,
Dr. John Araújo,
Dr. Plamen Ch. Ivanov.

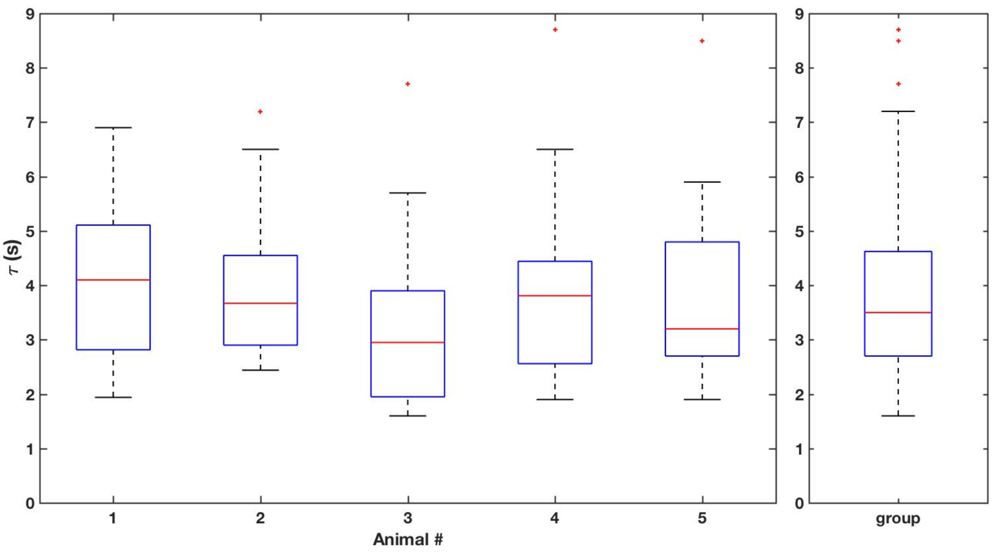

Figure 1: **Supplementary Figure-1: Statistics of τ period**. Panels (A) discloses descriptive statistic of τ period for 5 individual. Panel (B) display the group average for all 5 subjects. Panel (B) shows the group average for all events of all subjects show in Panel (A). Using the data of (A) we use ANOVA to test if the individuals show distinct τ period, we found no significant difference ($F(4, 106) = 0.949$, $p = 0.44$).

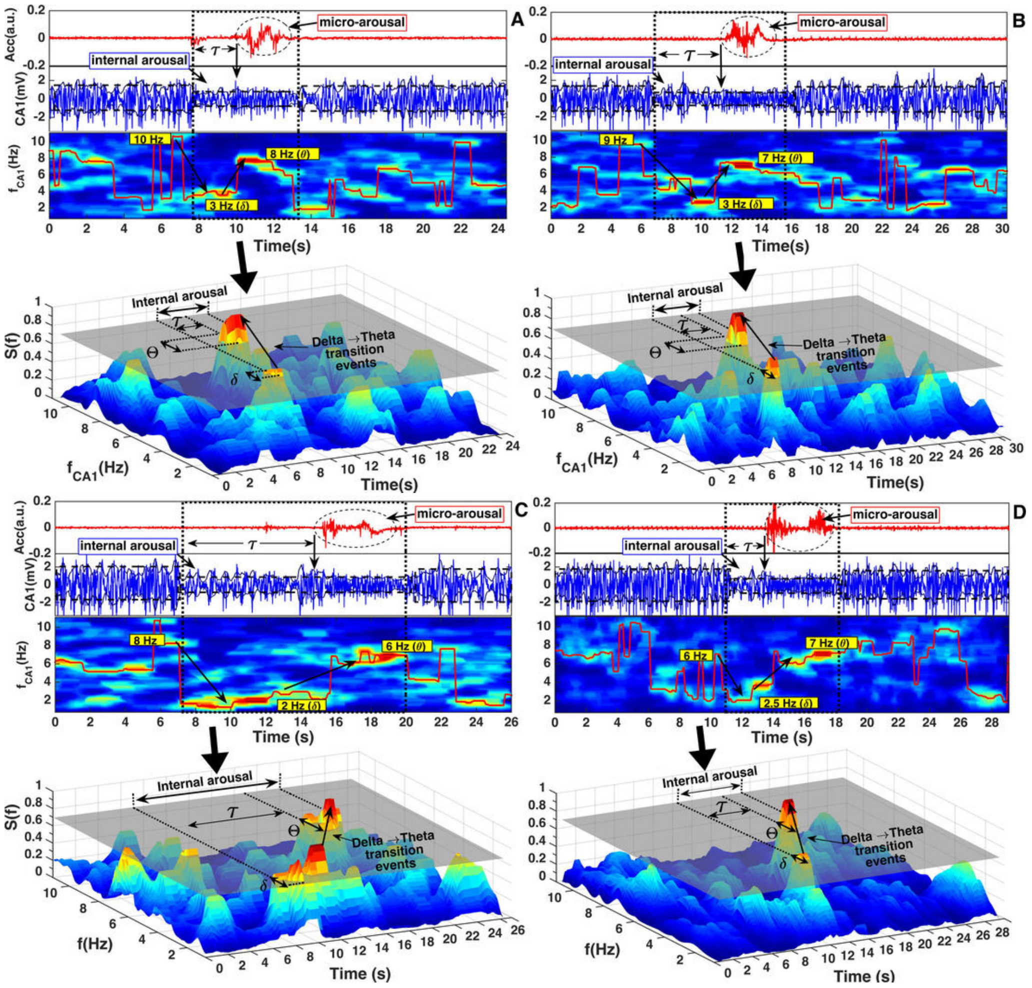

Figure 2: **Supplementary Figure-2. Summary of four individuals behavior where the hippocampus activity predicts micro-arousal in deep slow wave sleep**. For each one of four mice analyzed in this work, panel (a,b,c and d) display in the first plates the signature in the determinism signal [see Methods] obtained from the accelerometer (Acc) (red) and from electric probes at hippocampus CA1 sub-field (blue). The second plates depict the time series from Acc sensor (movement changes), placed on animals head (arbitrary units). The third plates evidence CA1 LFP time series (mV) (blue) and the Hilbert transform of the CA1 row signal (continuous dark blue lines) and its mean value. These LFP mean value were computed for the three time intervals separated by vertical dotted lines and are clearly different before and after internal arousal onset. The fourth plates display the spectrogram (Hz) obtained from CA1 data highlighting the $\theta \to \delta \to \theta$ transitions. Over all panels (a-d), vertical dotted lines indicate the beginning and the end of the changes in CA1 brain dynamics, which is named here as 'Internal arousal' (oblique-downwards and upward arrow in (c)).

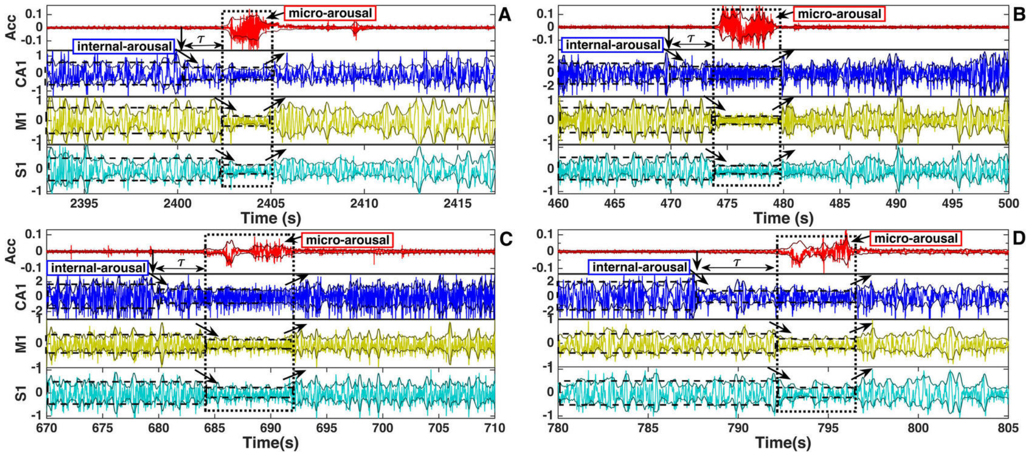

Figure 3: **Supplementary Figure-3. Brain dynamics are associated with micro arousal response - four mice results.** The first plate of Panels (a-d) shows the raw accelerometer (Acc) signal, in arbitrary units (a.u.) for each individuals analyzed (red curves), while second, third and fourth plates indicates the hippocampal local field potentials (in mV) of CA1 area (blue curves), primary motor area (M1) (yellow curves) or primary somatossensorial area (S1) (cyan curves), for the four individuals respectively; The envelope lines around CA1, M1 and S1 raw signals oscillations represent the output from Hilbert transform. Acc signal was calculated through obtaining the square root of the sum of X, Y and Z axis squared independently. Please note that CA1 area exhibits a change in dynamics (an intrinsic micro-arousal) around 3 seconds (τ) (mean value) before the initiation of critical muscle high amplitude activity in Acc. During the micro arousal motor activity period (highlighted by a dotted line in all graphs), also M1 and S1 areas exhibit a change in signal dynamics. This change in LFP dynamics is characterized by a reduction in average amplitude, firstly in hippocampus, after in cortex , as indicated by dashed lines and arrows in each case, and by changes in geometric figure colors in panel (e). Note also that after micro-arousal motor activity period all signal patterns from accelerometer and brain LFP average amplitudes return to the initial state, characterized by higher averages.

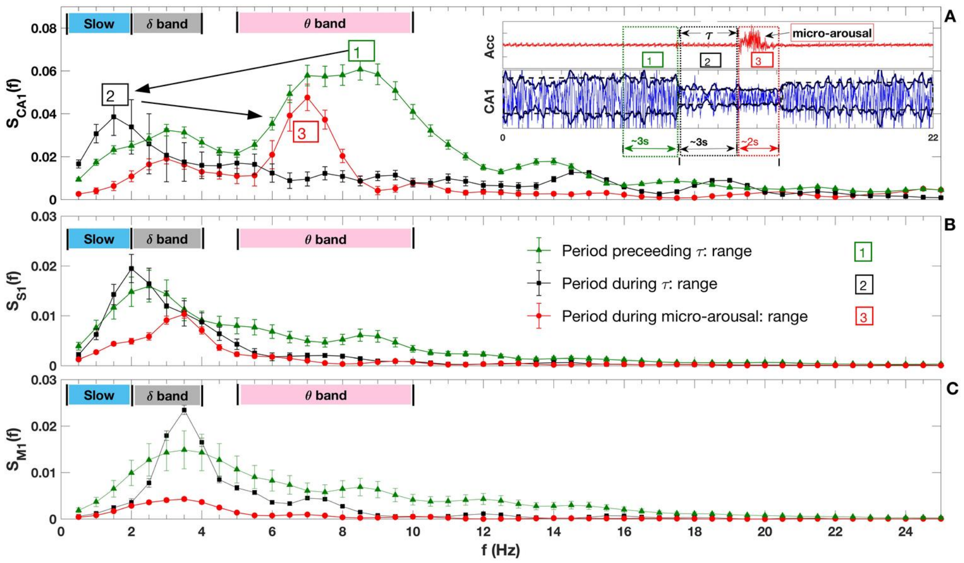

Figure 4: **Supplementary Figure-4. Hippocampal and cortical LFP power spectrum behavior related to micro-arousal.** Panel (A) display the mean power spectrum from the three time intervals with the transition between the dominant frequencies (arrows). The bars in the power spectra depicts the standard error. The inset panel shows a standard example of micro-arousal dynamics: ([1], green) period of time preceding the τ interval, ([2], black) period of time corresponding to the τ interval, and ([3], red) time interval corresponding to the micro-arousal events or post tau interval. Panel (B) shows the mean power spectrum of the three selected time intervals corresponding to pre-tau, tau and post-tau from LFP signal of cortical S1 brain area. The last, in panel (C), we observe the mean power spectrum of the same three selected time intervals corresponding to pre-tau, tau and post-tau, from LFP signal of cortical M1 brain area.
